# Off-grid hydroacoustic signal orientation estimation based on interpolation and subspace fitting in coprime arrays

**DOI:** 10.1371/journal.pone.0310415

**Published:** 2024-11-07

**Authors:** Chuanxi Xing, Guangzhi Tan, Qiang Meng, Yanling Ran, Mao Lu

**Affiliations:** 1 College of Electrical and Information Technology, Yunnan Minzu University, Kunming, Yunnan, China; 2 Yunnan Key Laboratory of Unmanned Autonomous System, Kunming, China; Whale Wave Technology Inc, CHINA

## Abstract

This letter presents a novel approach to sparse Bayesian underwater acoustic signal direction estimation. The proposed method incorporates interpolation of the coprime array and signal subspace fitting. It addresses the limitations of the hydrophone coprime array in utilizing all array elements’ information and mitigates the interference of ocean noise in shallow waters, which impairs the accuracy and resolution of target direction estimation. Firstly, the hydroacoustic signals are received using a coprime array, then the missing information is filled by interpolating the virtual array elements in the virtual domain, and by optimizing the design of the atomic norm and reconstructing the covariance matrix, the direction-of-arrival (DOA) estimation is performed using all the information of the received signal. Then, the received signal is reconstructed in conjunction with the reconstructed covariance signal subspace, which effectively reduces the impact of background noise. Finally, we derive an off-grid sparse model for the reconstructed signal by exploiting sparsity in the null domain and use Bayesian learning to compute the maximum a posteriori probability of the source signal, thus achieving DOA estimation. The results of numerical simulations and sea trial experimental data indicate that the use of subarrays comprising 5 and 3 array elements, respectively, is sufficient to effectively estimate 12 source angles. Furthermore, the estimation of the DOA can be accurately carried out under low signal-to-noise ratio conditions. This method effectively utilizes the degrees of freedom provided by the virtual array, reducing noise interference, and exhibiting better performance in terms of positioning accuracy and algorithm stability.

## 1 Introduction

Direction-of-arrival (DOA) estimation is one of the fundamental techniques in the field of hydroacoustic signal processing [1]. Conventional DOA estimation algorithms in the field of hydroacoustic localization include conventional beamforming (CBF) [2], minimum variance distortion-free response (MVDR) [3], and multiple signal classification (MUSIC) [4] estimation of signal parameters via rotational invariance techniques (ESPRIT) [5] subspace algorithms with super-resolution capability subspace algorithms with super-resolution capability. However, a large number of snapshots are needed to ensure that the sampling covariance matrix is close to the real value, and in unfavorable environments such as low signal-to-noise ratios, the performance of these algorithms in estimating the target orientation can be significantly reduced [6,7]. To enhance the efficacy and resilience of the algorithm in environments with weak signals, the sparse Bayesian learning (SBL) method has been proposed as a means of accurate bearing estimation in the presence of Gaussian white noise [8,9]. In recent developments, various off-grid methods utilizing SBL have emerged. Among these, Yang et al. introduced the off-grid sparse Bayesian inference (OGSBI) technique, specifically designed for 1D DOA estimation with linear arrays [10].

In general, uniform linear arrays (ULAs) are employed in the aforementioned methods. Nevertheless, the high redundancy inherent to ULA has prompted the development of sparse array designs to reduce redundancy. In recent years, there has been a great deal of interest in the systematic design of sparse arrays, which are known as coprime arrays [11–13]. It can be demonstrated that coprime arrays offer greater apertures and degrees of freedom (DOFs) than ULAs. This suggests that DOA estimation with coprime arrays is likely to be highly effective. The prevailing DOA estimation algorithms, which employ coprime arrays, are designed to derive augmented virtual arrays and manipulate the corresponding virtual array signals for DOA retrieval [14–16]. However, since the coprime arrays are partially incrementable arrays, there are voids in their different mutual prime arrays, which leads to discontinuities in the derived virtual arrays. In the domain of based DOA estimation methods applied in coprime arrays, a common solution is to extract the maximum continuous segments for subsequent coprime array signal processing, such as spatial smoothing MUSIC (SS-MUSIC) [17,18] and covariance matrix sparse reconstruction [19]. It is evident that the performance of the array is diminished to some extent as a consequence of the removal of the discontinuous virtual array elements. In order to address this issue, an interpolated array element algorithm was proposed in the literature [20]. However, in terms of both sparsity and accuracy, the off-grid sparse Bayesian algorithm is a more advantageous solution.

In order to address the aforementioned issues, this paper proposes an off-grid sparse Bayesian inference algorithm based on the interpolated value subspace reconstruction of signals from the coprime array (ICO-SF-OGSBI). Firstly, the missing holes of the virtual domain of the mutual mass array are filled to form a continuous virtual line array. Secondly, the signals are reconstructed by the optimal design of the atomic paradigm minimization and the interpolation of the covariance matrix signal subspace of the virtual array. Finally, the signals are reconstructed by using the off-grid sparse Bayesian inference algorithm for DOA estimation. The proposed method enhances the array aperture and degrees of freedom, thereby mitigating the impact of missing information and predefined grid points in the virtual domain. Furthermore, it demonstrates robust direction-of-arrival estimation performance even in the presence of low signal-to-noise ratios. Numerical simulation results and sea trial data validation reflect the effectiveness of the algorithm. When the signal-to-noise ratio (SNR) is -20 dB and the number of snapshots is 256, the estimation accuracy of the proposed algorithm is enhanced by 58.29% in comparison to the method described in the literature [20].

## 2 Coprime array model

**Fig 1:**
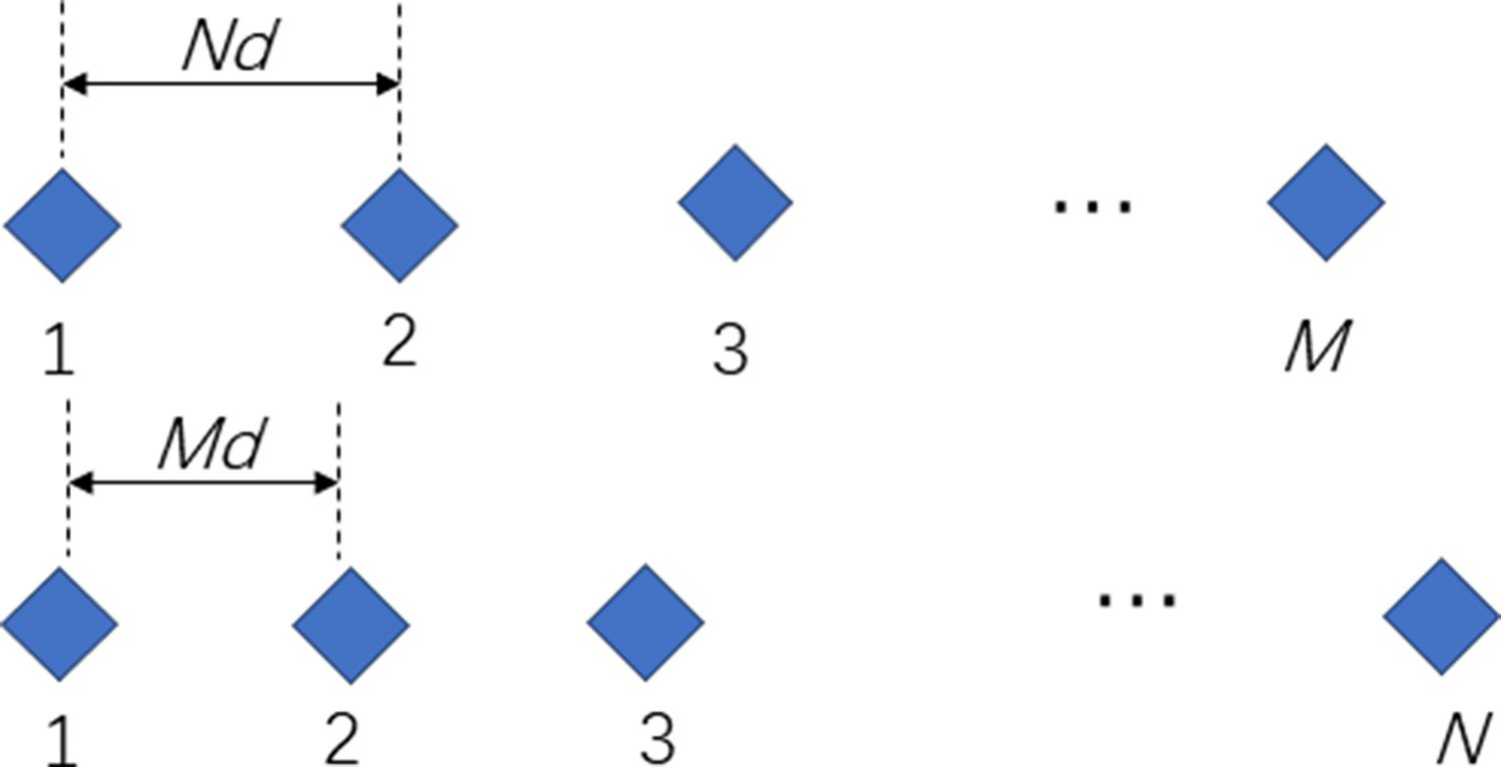
Coprime array.

A coprime array is defined as a concatenation of a pair of sparse uniform arrays, with the array elements located at {0,Md,2Md,…,(N−1)Md} and {0,Nd,2Nd,…,(M−1)Nd}, where *M* and *N* are mutually prime integers, *d* is equal to the half wavelength and is *d* = *λ*/2, as shown in Fig 1. In accordance with the principles of coprimality [21], the coprime array

S={Mnd|0≤n≤N−1}∪{Nmd|0≤m≤M−1}, (1)

where S is a non-uniform linear array containing |S|=M+N−1 array elements with an aperture max((N−1)Md,(M−1)Nd), where |⋅| denotes the number of elements of the set.

In light of the existence of non-correlated, narrowband signal sources in space with the direction of arrival of *θ*_*k*_,*k* = 1,2,…,*K*, incident on the coprime array S, the received signal vector of the array at the time of *t* can be modelled as

$$y_{S}(t)=\sum_{k=1}^{K}a_{S}(\theta_{k})s_{k}(t)+n_{S}(t)=A_{S}s(t)+n_{S}(t),$$ (2)

where $A_{S}=[a_{S}(\theta_{1}),a_{S}(\theta_{2}),\ldots ,a_{S}(\theta_{K})]\in {\mathbb{C}}^{|S|\times K}$ is the coprime array manifold matrix in column *k*, $s(t)={\left[s_{1}(t),s_{2}(t),\ldots ,s_{K}(t)\right]}^{T}$ is the signal waveform vector, $n_{S}(t)$ is the Gaussian white noise, and [⋅]^T^ denotes the transpose. Where $a_{S}(\theta_{k})$ denotes the coprime array steering vector corresponding to the *k*th incident signal source ***θ***_***k***_, which can be expressed as

$$a_{S}(\theta_{k})={\left[1,e^{-j\frac{2\pi }{\lambda }\mu_{2}\sin(\theta_{k})},\ldots ,e^{-j\frac{2\pi }{\lambda }\mu_{\left|S\right|}\sin(\theta_{k})}\right]}^{T},$$ (3)

where *j* denotes the imaginary part unit which satisfies $j=\sqrt{-1}$; $\mu_{i}\in S,i=1,2,\ldots ,M+N-1,$ is the actual position of the physical array element in the reciprocal array, the first physical array element is placed at the zero position, i.e. *μ*_1_ = 0.

The signal covariance matrix of the array received signal $y_{S}(k)$ can be calculated as

$${\hat{R}}_{y_{S}y_{S}}=\frac{1}{T}\sum_{t=1}^{T}y_{S}(t){y_{S}}^{H}(t).$$ (4)

Here, ${\hat{R}}_{y_{S}y_{S}}$ represents a maximum likelihood estimate of the received signal covariance. When the number of sampling beats T tends to infinity with a smooth traversal, the proximity covariance matrix converges to the ideal covariance matrix ***R***. Nevertheless, when the number of snapshots is constrained, the discrepancy between ${\hat{R}}_{y_{S}y_{S}}$ and ***R*** will negatively impact the efficacy of the DOA estimation.

## 3. Subspace fitting of interpolated coprime arrays

To meet the requirements of the off-grid sparse Bayesian inference algorithm for uniform array signals, the virtual domains of the coprime arrays were interpolated, and uniform arrays were constructed. The covariance matrix of the constructed uniform array signal was then obtained using Eq (4). To address the potential issue of a missing covariance matrix rank caused by interpolation, an optimization method was designed to determine the atomic paradigm number for the reconstruction of the covariance matrix (interpolated virtual array). Subsequently, eigenvalue decomposition was performed on the reconstructed covariance matrix to obtain a signal subspace. This signal subspace was then used to reconstruct the signal, thereby reducing the effects of noise.

**Fig 2:**
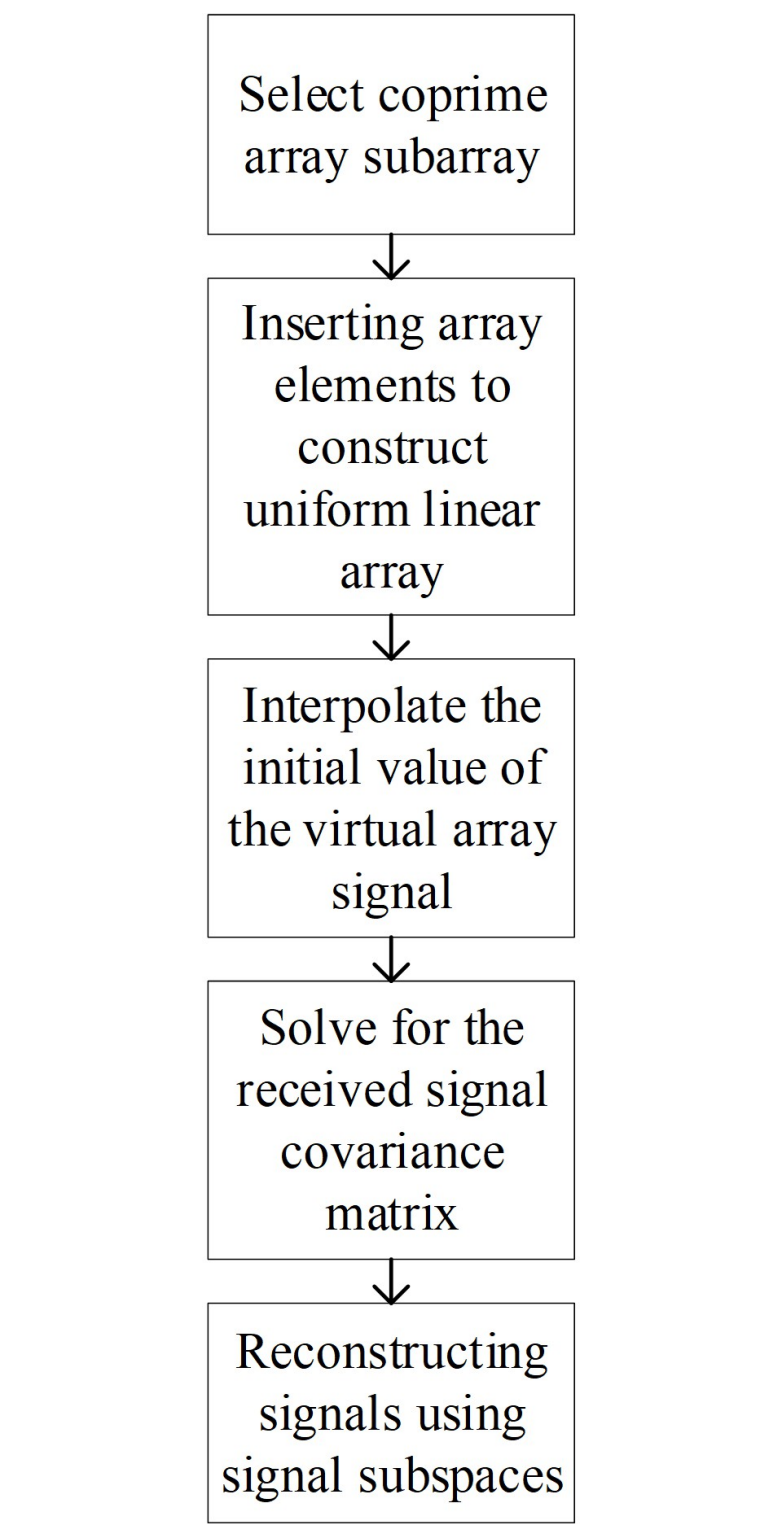
Flowchart of the subspace fitting algorithm for interpolated coprime arrays.

The flowchart of the subspace fitting algorithm for the interpolated coprime array is presented in Fig 2.

### 3.1 Interpolation model for coprime arrays

**Fig 3:**
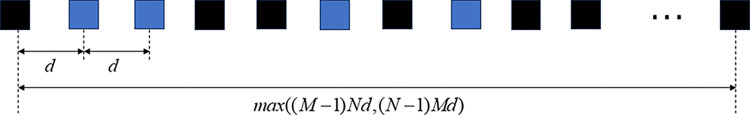
Coprime array with interpolated array elements.

A coprime array provides systematic array configurations for sparse sensing. However, their inhomogeneity limits the adoption of traditional DOA estimation methods. To overcome the problem, as shown in Fig 3, a uniform linear array is created by inserting virtual array elements between the physical array elements of the coprime array. These virtual array elements are located at integral multiples of half the wavelength of the physical array elements. The resulting interpolated ULA can be expressed as

V={ld|0≤=ld≤max(S),l∈ℤ}, (5)

where V has the same aperture as the coprime array, where *l*∈ℤ is a consecutive integer and S⊂V.

The blue squares in Fig 3 indicate interpolated array elements that exist in a mathematical rather than a physical sense. This suggests that the corresponding received signals are actually unknown.

The received signal of an interpolated coprime array can be expressed as

$${\langle y_{V}(k)\rangle }_{l}=\{\begin{array}{l} {\langle y_{S}(k)\rangle }_{l},ld\in S,\\ 0,\;ld\in V\backslash S, \end{array}$$ (6)

where 〈⋅〉_*l*_ denotes the element corresponding to the array element located at *ld* and V\S denotes the interpolated array element. Accordingly, the elements of the true array element are defined as 1, while the elements of the interpolated array element are defined as 0, i.e.


$${\langle g\rangle }_{l}=\{\begin{array}{l} 1,\;ld\in S,\\ 0,ld\in V\backslash S. \end{array}$$ (7)

The initialization signal of the array $y_{V}$ can be represented by $y_{S}$, i.e.

$$y_{V}(t)=y_{S}(t)⊙g,$$ (8)

where ⊙ denotes the Hadamard product operator. The theoretical received signal of the array can be modeled as

$$y_{V}(t)=\sum_{k=1}^{K}a_{V}(\theta_{k})s_{k}(t)+n_{V}(t)=A_{V}s(t)+n_{V}(t).$$ (9)

here

$$a_{V}(\theta_{k})={\left[1,e^{-j\frac{2\pi }{\lambda }u_{2}\sin(\theta_{k})},\ldots ,e^{-j\frac{2\pi }{\lambda }u_{\left|U\right|}\sin(\theta_{k})}\right]}^{T},$$ (10)

where $a_{V}(\theta_{k})$ is a vector of orientations corresponding to the interpolated ULA of the *k*th source signal *θ*_*k*_ and $A_{V}=[a_{V}(\theta_{1}),a_{V}(\theta_{2}),\ldots ,a_{V}(\theta_{K})]\in {\mathbb{C}}^{|V|\times K},$ where $u_{v}\in V,v=1,2,\ldots ,|V|,$ denotes the position of the *v*th array element in the interpolated ULA, *μ*_1_ = 0, and $n_{V}(t)\in {\mathbb{C}}^{\left|V\right|}∼CN(0,\sigma_{n}^{2}I)$ are vectors of additive Gaussian white noise corresponding to |U|.

Then the signal covariance matrix of the array received signal $y_{V}(k)$ can be calculated as

$${\hat{R}}_{y_{V}y_{V}}=\frac{1}{T}\sum_{t=1}^{T}y_{V}(t)y_{V}^{H}(t).$$ (11)

The theoretical covariance matrix corresponding to the uncorrelated signals received at ULA has a Hermitian Toeplitz structure. This property can be exploited a priori to perform structured matrix recovery. Conversely, the noiseless covariance matrix also exhibits the low-rank property due to the relatively small number of event sources compared to the number of array elements in the interpolating array. Consequently, the covariance matrix of the interpolated array can be recovered by solving the following optimization problem, i.e.

$$\begin{array}{l} \underset{z}{\min}\;rank(T(z)),\\ \mathrm{subject}\;\mathrm{to}\;\begin{array}{c} \|(T(z)-{\hat{R}}_{y_{V}y_{V}})⊙G{\|}_{F}^{2}\leq \delta ,\\ T(z)\geq 0, \end{array} \end{array}$$ (12)

where *G* = *gg*^T^ is a |V|×|V| dimensional binary matrix used to distinguish between known (non-zero) and unknown (zero) elements of the initialized sample covariance matrix ${\hat{R}}_{y_{V}y_{V}}$. T(z)≥0 denotes the Hermitian PSD Toeplitz matrix, where $z\in {\mathbb{C}}^{\left|U\right|}$ serves as the first column element, rank(⋅) denotes the rank of the matrix, ‖⋅‖_*F*_ denotes the Frobenius norm, and *δ* is a user-defined parameter to constrain the fitting error.

The introduction of a convex relaxation of the kernel paradigm enables the NP-hard rank minimization problem (12) to be reformulated.

$$\begin{array}{l} \min\|(T(z)-{\hat{R}}_{y_{V}y_{V}})⊙G{\|}_{F}^{2}+\xi \|T(z){\|}_{*},\\ \mathrm{subject}\;to\;T(z)\geq 0, \end{array}$$ (13)

where ‖⋅‖_*_ denotes the kernel norm of the matrix and *ξ* is the regularization parameter that balances the fitting error and the kernel norm. Since

$$\|T(z){\|}_{*}=\mathrm{Tr}(\sqrt{T^{H}(z)T(z)}),$$ (14)

where Tr(⋅) denotes the trace operator, and the PSD constraint on T(z) converts the kernel-paradigm minimization problem (16) equivalently to a trace minimization problem, i.e.


$$\begin{array}{l} \underset{z}{\min}\;\|(T(z)-{\hat{R}}_{y_{V}y_{V}})⊙G{\|}_{F}^{2}+\xi \mathrm{Tr}(T(z)),\\ \mathrm{subject}\;\mathrm{to}\;T(z)\geq 0. \end{array}$$ (15)

Problem (15) is a convex optimization problem which can be solved efficiently by interior point method. The optimized covariance matrix $T(\hat{z})\in {\mathbb{C}}^{\left|U|\times |U\right|}$ is an estimate of ${\hat{R}}_{y_{U}y_{U}}$, i.e. the covariance matrix of the signal received by the interpolating ULA, and the number of DOFS achievable |S| to |V|.

### 3.2 Reconstruction of received signal using signal subspace

The estimated covariance matrix is decomposed in order to obtain the signal subspace matrix. This is because the signal subspace contains the main DOA information for the array, which can be used to reconstruct the signal. Finally, the signal DOA is solved by off-grid sparse Bayesian inference.

The optimized covariance matrix $T(\hat{z})$ is to be decomposed into its eigenvalues. i.e.

$$T=\sum_{i=1}^{D}b_{i}u_{i}u_{i}^{H}+\delta_{n}^{2}\sum_{i=D+1}^{L}b_{i}u_{i}u_{i}^{H}=U_{s}\Lambda_{s}U_{s}^{H}+\delta_{n}^{2}U_{n}U_{n}^{H},$$ (16)

where $b_{1}\geq b_{2}\geq \ldots \geq b_{D+1}=\ldots =b_{L}^{2}=\delta_{n}^{2};$ $\Lambda_{s}=diag(b_{1},b_{2},\ldots ,b_{D})$. It can be demonstrated that equality *D* = *K* is only valid if the signals in question are two-by-two independent of each other. In this case, the eigenvectors $U_{s}=[u_{1},u_{2},\ldots ,u_{K}]$ corresponding to the first *K* eigenvalues constitute the signal subspace and the remaining *L*−*K* eigenvectors $U_{n}=[u_{D+1},u_{D+2},\ldots ,u_{L}]$ constitute the noise subspace. In the event that the signals are uncorrelated, in accordance with the relationship between the signal subspace and the space tensored by the array of flow-oriented vectors, it can be demonstrated that $span(U_{s})=span(A(\theta ))$. Consequently, there exists and exists only one ***C*** such that it satisfies, i.e.


$$U_{s}=A(\theta )C.$$ (17)

The Eq (16) yields the result

$$T=A(\theta )R_{s}A^{H}(\theta )+\delta_{n}^{2}I=U_{s}\Lambda_{s}U_{s}^{H}+\delta_{n}^{2}(I-U_{s}U_{s}^{H}),$$ (18)

assume

$$C=R_{s}A^{H}(\theta )U_{s}{\left(\Lambda_{s}-\delta_{n}^{2}I\right)}^{-1}.$$ (19)

However, in practice, due to the existence of various errors in the array as well as the effect of multipath and other factors, the signal subspace and the subspace formed by the array flow pattern tensor are not equal in the strict sense, so Eq (19) does not hold. In order to resolve this issue, a fitting equation can be constructed to ensure that Eq (19) holds, i.e.

$$\theta ,C=\min∥U_{s}W‐A(\theta )C{∥}_{F}^{2},$$ (20)

where $∥\cdot {∥}_{F}^{2}$ denotes the square of the Frobenius norm of the matrix and the optimal weighting matrix $W={\left(U_{s}-\delta_{n}^{2}I\right)}^{2}\Lambda_{s}^{-1}$.

Due to the presence of noise, the relationship between the signal subspace in Eq (17) and the space consisting of the array flow pattern is redefined as

$${\bar{U}}_{s}=A(\theta )C+E,$$ (21)

where ${\bar{U}}_{s}=WU_{s}$, in this paper, the weighting matrix consists of the eigenvalues of the signal generated during the eigendecomposition of the covariance matrix, $W=\Lambda_{s}=diag(l_{1},l_{2},\ldots ,l_{D})$; ***E*** is the complex Gaussian distributed noise matrix obeying a zero mean.

## 4. Off-grid sparse Bayesian inference algorithm

The spatial angular range [-*π*/2,*π*/2] is uniformly divided into *N* grid points, each of which represents a possible incidence direction, i.e. $\hat{\theta }=\{{\hat{\theta }}_{1},{\hat{\theta }}_{2},\ldots ,{\hat{\theta }}_{N}\}$, and *K*<*M*≪*N*, from which the grid spacing $r=\{{\hat{\theta }}_{2}-{\hat{\theta }}_{1},{\hat{\theta }}_{3}-{\hat{\theta }}_{2},\ldots ,{\hat{\theta }}_{N}-{\hat{\theta }}_{N-1}\}$ can be known. Obviously, the target orientation can be converted to an overcomplete sparse representation in the divided *N* grid points. In the off-grid sparse model, the problem of mismatch between the target incidence direction and the grid points is faced, i.e., ${\hat{\theta }}_{k}\notin \{{\hat{\theta }}_{1},{\hat{\theta }}_{2},\ldots ,{\hat{\theta }}_{N}\}$ [9]. Generally adopting denser grid points can reduce the error, but this does not include all the possible incidence directions and increases the amount of computation. To address this problem, the off-grid error is introduced into the array’s prevalence matrix by doing a first-order Taylor expansion between two neighbouring grid points, and the guidance vector is approximated as

$$\varphi (\theta_{k})\approx a({\hat{\theta }}_{n_{k}})+b({\hat{\theta }}_{n_{k}})(\theta_{k}-{\hat{\theta }}_{n_{k}}),$$ (22)

where ${\hat{\theta }}_{n_{k}}$ denotes the closest mesh point to *θ*_*k*_, *n*_*k*_∈{1,2,⋯,*N*} $b({\hat{\theta }}_{n_{k}})=a'({\hat{\theta }}_{n_{k}})$. We let $\beta ={\left[\beta_{1},\beta_{2},\cdots ,\beta_{N}\right]}^{T}\in {\left[-r/2,r/2\right]}^{N}$, the mesh error can be expressed as

$$\beta_{n}=\{\begin{array}{l} \theta_{k}-{\hat{\theta }}_{n_{k}},\;\hat{S}\neq 0,n=n_{k}\\ 0,\;\hat{S}=0,n\neq n_{k} \end{array}$$ (23)

According to Eq (22), the overcomplete array prevalence matrix can be represented as[10]

$$\Phi ={\hat{A}}_{\hat{\theta }}+Bdiag\{\beta \},$$ (24)

where ${\hat{A}}_{\hat{\theta }}=[a({\hat{\theta }}_{1}),a({\hat{\theta }}_{2}),\ldots ,a({\hat{\theta }}_{N})]$, $B=[b({\hat{\theta }}_{1}),b({\hat{\theta }}_{2}),\ldots ,b({\hat{\theta }}_{N})]$. By introducing the mesh error, Eq (21) can be changed to

$${\bar{U}}_{s}=\Phi (\beta )\hat{C}+E,$$ (25)

where $\hat{C}$ is the new transform matrix. In the matrix $\hat{C}$, only the rows corresponding to the true incident direction of the source signal are non-zero and have values equal to the values of the corresponding rows of the original transformation matrix $\hat{C}$, while the row vectors corresponding to the non-incident signals are zero. In the matrix $\hat{C}$ there are only a small number of non-zero rows, thus exhibiting sparsity, and a compressed perception algorithm can then be introduced.

The estimation of hydroacoustic target orientation is carried out using Bayesian inference, and the optimal estimate can be obtained. Assuming that the noise signal obeys a complex Gaussian distribution, the likelihood function about $\hat{C}$ is

$$p({\bar{U}}_{s}|\hat{C};\alpha_{0})=CN({\bar{U}}_{s}|\Phi \hat{C},{\alpha_{0}}^{-1}),$$ (26)

where CN denotes the complex Gaussian distribution, *α*_0_ = *σ*^−2^, where *σ*^2^ is the noise variance, *α*_0_ is usually unknown, and is assumed to follow a Gamma prior distribution. Let the sparse signal ***Γ*** be

$$p(\hat{C}|\gamma )=CN(\hat{C}|0,\Gamma ),$$ (27)

where ***γ*** is a set of hyperparameters, ***γ*** = [*γ*_1_,*γ*_2_,⋯,*γ*_*N*_]^T^, denotes the power of the source signal incident to the array in each direction, which affects the sparsity of the sparse signal. Where ***Γ*** = diag(***γ***) denotes the covariance matrix of the sparse signal.

Combining the a priori information and the likelihood function, the joint probability density function is obtained as

$$\begin{array}{l} \;p(\hat{C},{\bar{U}}_{s},\alpha_{0},\gamma ,\beta )\\ =p({\bar{U}}_{s}|\hat{C},\alpha_{0},\beta )p(\hat{C}|\gamma )p(\alpha_{0})p(\gamma )p(\beta ). \end{array}$$ (28)

The posterior probability density function of ${\bar{U}}_{s}$ is obtained from the Bayesian derivation as

$$\begin{array}{l} \;p(\hat{C}|{\bar{U}}_{s},\alpha_{0},\gamma ,\beta )\\ =\frac{p({\bar{U}}_{s}|\hat{C},\alpha_{0},\beta )p(\hat{C}|\gamma )}{p({\bar{U}}_{s}|\alpha_{0},\gamma ,\beta )}\\ =CN(\hat{C}|\mu ,\Sigma ), \end{array}$$ (29)

where the a posteriori mean and a posteriori covariance matrices of the sparse signals are respectively

$$\mu =\alpha_{0}\Sigma \Phi^{H}{\bar{U}}_{s},$$ (30)

$$\Sigma ={\left(\alpha_{0}\Phi^{H}\Phi +\Gamma^{‐1}\right)}^{‐1},$$ (31)

where the computation of ***μ*** and ***Σ*** requires the estimation of hyperparameters, *α*_0_ ***γ*** and ***β***. In this paper, maximum a posteriori probability estimation is used to estimate these hyperparameters, i.e., by maximizing $p(\alpha_{0},\gamma ,\beta |\hat{C})$, *α*_0_ and ***γ*** The two parameters are given by the following equation

$${\alpha_{0}}^{new}=\frac{TM+c-1}{d+\sum_{t=1}^{T}{\left\|{\bar{U}}_{s}(t)-\Phi \mu (t)\right\|}_{2}^{2}+Ttr(\Phi^{H}\Sigma \Phi )},$$ (32)

$$\gamma_{n}^{new}=\frac{‐T+\sqrt{T^{2}+4\rho \sum_{t=1}^{T}{\left[\Xi_{t}\right]}_{n}}}{2\rho },$$ (33)

where $\Xi_{t}≜\mu (t){\left(\mu (t)\right)}^{H}$ *c*,*d*→0, *ρ* is a positive constraint that takes a small value.

The off-grid error ***β*** can determine the accuracy of the target direction estimate. We can use the expectation maximization criterion to find the mesh error such that the expectation $E\{\ln[p({\hat{X}}_{SV}|\hat{C},\alpha_{0},\beta )p(\beta )]\}$ is maximized, which is equivalent to minimizing $E\{{\left\|{\bar{U}}_{s}-\Phi \hat{C}\right\|}_{2}^{2}\}$, i.e.

$$\begin{array}{l} E\{{\left\|{\bar{U}}_{s}-\Phi \hat{C}\right\|}_{2}^{2}\}=E\{{\left\|{\bar{U}}_{s}-({\hat{A}}_{\tilde{\theta }}+Bdiag\{\beta \})\hat{C}\right\|}_{2}^{2}\}\\ \;=\beta^{T}P\beta -2v^{T}\beta +F, \end{array}$$ (34)

where ***F*** is a constant depending on ***β***. ***P*** is a semi-positive definite matrix with the expression

$$P=ℜ\{\bar{B^{H}B}⊙(\mu \mu^{H}+\Sigma )\},$$ (35)

$$v=ℜ\{\mathrm{diag}(\bar{\mu })B^{H}({\bar{U}}_{s}-{\hat{A}}_{\hat{\theta }}\mu )-\mathrm{diag}(B^{H}{\hat{A}}_{\hat{\theta }}\Sigma )\}.$$ (36)

The angular correction vector is obtained from the above derivation as[22]

$$\beta^{new}=\arg\underset{\beta \in {\left[-\frac{r}{2},\frac{r}{2}\right]}^{N}}{\min}\{\beta^{T}P\beta -2v^{T}\beta \}.$$ (37)

From this point on, by differentiating with respect to ***β*** using Eq (34) and setting the derivative equal to zero, the expression for ***β*** can be obtained as

$${\hat{\beta }}_{n}=\frac{v_{n}-{\left(P_{n}\right)}_{‐n}^{T}\beta_{‐n}}{P_{n}},$$ (38)

where ***β***_***-n***_ is ***β*** without the *n*th vector ***β***. With the constraint $\beta_{n}\in [-r/2,r/2]$, we can get

$${\hat{\beta }}_{n}^{\mathrm{new}}=\{\begin{array}{l} {\hat{\beta }}_{n},\;{\hat{\beta }}_{n}\in [‐\frac{r}{2},\frac{r}{2}]\\ \frac{r}{2},\;{\hat{\beta }}_{n}>\frac{r}{2}\\ ‐\frac{r}{2},\;{\hat{\beta }}_{n}<‐\frac{r}{2} \end{array}$$ (39)

In the above Bayesian inference, we maximize the posterior probability to find the update formulae for the two hyperparameters *α*_0_ and ***γ***. Then, using these two hyperparameters, we find the update formulae for the hyperparameters of the angular correction vectors using the expectation maximization criterion ***β*** and obtain the final mesh error *β*_*n*_. Finally, we initialize *α*_0_, ***γ*** and ***β*** and keep iterating Eqs (32), (33) and (37) until convergence, so that we can compute the estimates *K* of the incident signal angles i.e., $\theta_{k}=\theta_{n_{k}}+{\hat{\beta }}_{n_{k}}$, *k* = 1,2,⋯,*K*.

The specific procedure of the off-grid sparse Bayesian inference algorithm based on interpolation and subspace fitting within the coprime array is as follows.

1. The coprime array subarrays are selected to be M and N, respectively.
2. The process of interpolation is employed to fill the gaps in the coprime array, thus enabling the construction of a uniform array that is capable of receiving signals.
3. The covariance matrix of the resulting received signal is obtained, and the received signal is reconstructed using the signal subspace of the covariance matrix.
4. Construct an off-grid sparse model to obtain an overcomplete sparse dictionary.
5. Initialize the noise and signal hyperparameters *α*_0_ and ***γ*** respectively, and initialize the angle correction vector ***β***, the mean ***μ*** and the variance ***Σ*** to zero.
6. Eqs (30) and (31) were used to solve for mean and variance, respectively.
7. The updated hyperparameters $\alpha_{0}^{\mathrm{new}}$, ***γ***^new^ and ***β***^new^ are obtained using Eqs (32), (33) and (37).
8. When ${\left\|\gamma^{n}-\gamma^{n-1}\right\|}_{2}/{\left\|\gamma^{n-1}\right\|}_{2}\leq \tau$ or to the maximum number of iterations, continue to the next step; if it does not converge, skip to step 6.
9. Calculate the DOA for the target.

## 5. Experimental performance and simulation

The objective of this section is to perform a theoretical calibration using numerical simulations in order to guarantee the accuracy of our theoretical derivations and the validity of our algorithms. The specific parameter settings of the algorithm proposed in this paper are presented in Table 1 for reference. In order to further verify the superiority of the proposed algorithms, a comparative analysis is conducted with the MUSIC algorithm based on the coprime array (CO-MUSIC), the root-MUSIC algorithm based on the coprime array (CO-ROOT-MUSIC) [20], the OGSBI algorithm based on an uniform linear array (ULA-OGSBI) [10], and the SS-MUSIC algorithm based on a coprime array (CO-SS-MUSIC) [17]. As proposed in the literature [20], the CO-MUSIC and CO-ROOTMUSIC algorithms form a homogeneous array through interpolation in a coprime array. Subsequently, the authors employ the MUSIC algorithms in conjunction with the ROOT-MUSIC algorithm and the MUSIC algorithm for the estimation of direction of arrival (DOA). The CO-SS-MUSIC algorithm is vectorised by the covariance matrix, then reconstructed using a spatial smoothing method, and finally employs the MUSIC algorithm for DOA estimation [18].

**Table 1:** Algorithmic experimental parameter design.

Parameter name	Parameter value
CVX regularization parameter OGSBI regularization parameter Iterated residual threshold Maximum iterations	0.25 10^−2^ 10^−3^ 500
mean ***μ*** variance ***Σ***	0 0

Unless otherwise specified, the following parameters and definitions are employed in our numerical simulations: a setup of *M* = 3, *N* = 5 for the number of subarrays of the coprime array, with the spacing of the arrays set to half the wavelength, i.e., *d* = 0.5m, and the spatial angle divided from -90 to 90 degrees. The center of the source is considered to emit at a frequency of 1000 Hz. The signal-to-noise ratio is defined in accordance with the following equation: SNR = 10lg(*P*_*s*_/*P*_*n*_). The error is quantified using the root mean square error (RMSE), defined as

$$\mathrm{RMSE}=\sqrt{\frac{1}{SK}\sum_{s=1}^{S}\sum_{k=1}^{K}{\left({\hat{\theta }}_{k_{s}}-\theta_{k}\right)}^{2}},$$ (40)

where *S* is the total number of independent Monte Carlo experiments and ${\hat{\theta }}_{k_{s}}$ is the orientation estimate of the *s* Monte Carlo experiment in the *k* source.

### 5.1 Maximum number of recognisable sources

**Fig 4:**
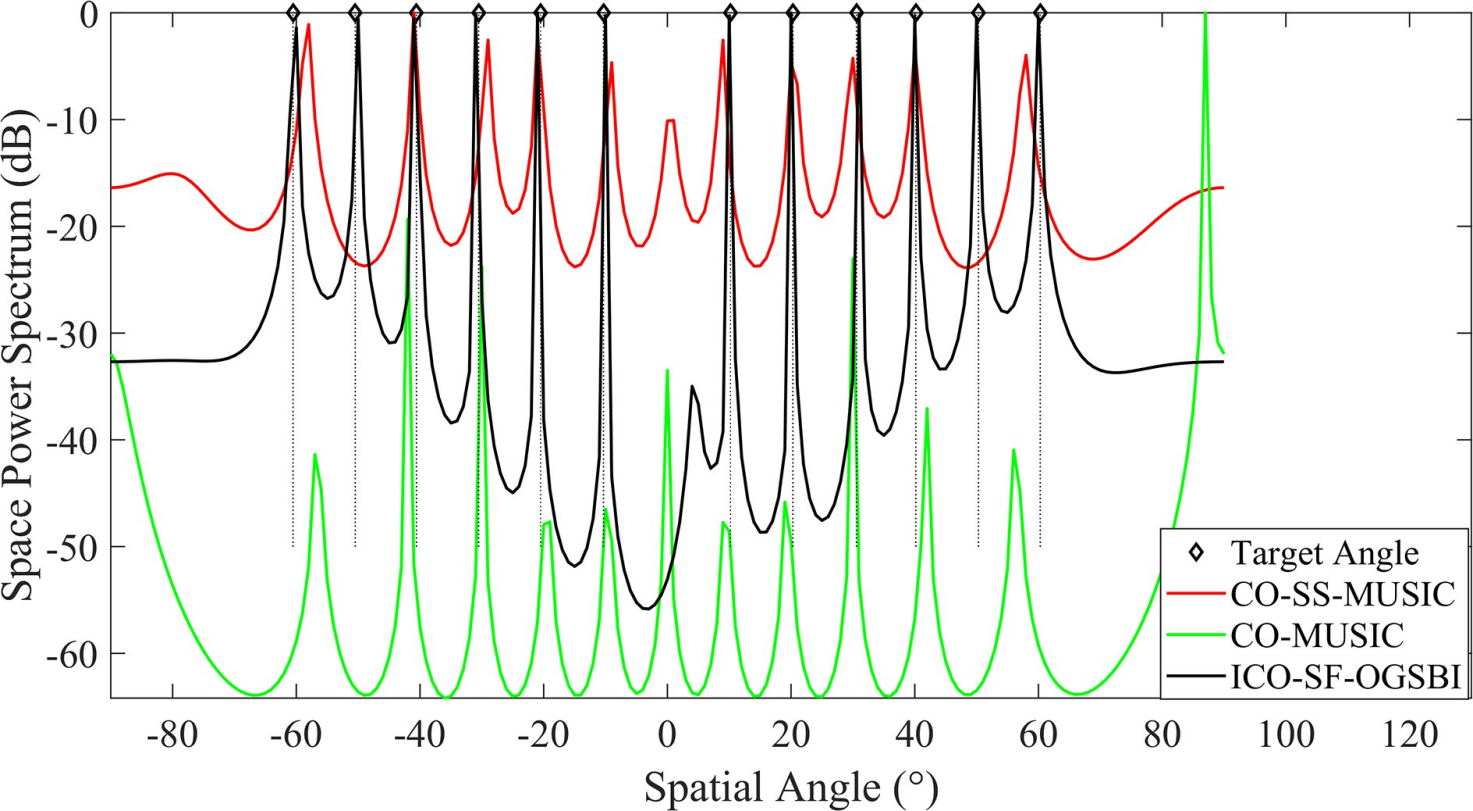
Angle estimation of 12 incident sources by the interface array.

In order to further validate the growth of the algorithm’s degrees of freedom, 12 incident sources distributed in the angular range of [‐60.5°‐50.5°‐40.6°‐30.5°‐20.5°‐10.3°10.2°20.3°30.6°40.2°50.3°60.3°], i.e., *K* = 12, are established in the space. The input signal-to-noise ratio and angular spacing of the array elements are *r* = 1. The number of sampled snapshots is defined as *T* = 200. With regard to the angular estimation of these 12 sources, our method is highly efficient, as illustrated in Fig 4. A comparison of the proposed method with the CO-MUSIC algorithm and the CO-SS-MUSIC algorithm reveals that the former makes more effective use of the degrees of freedom provided by the virtual array and estimates the 12 incident sources with a narrow main flap width.

### 5.2 Spatial spectrum and root mean square error analysis

**Fig 5:**
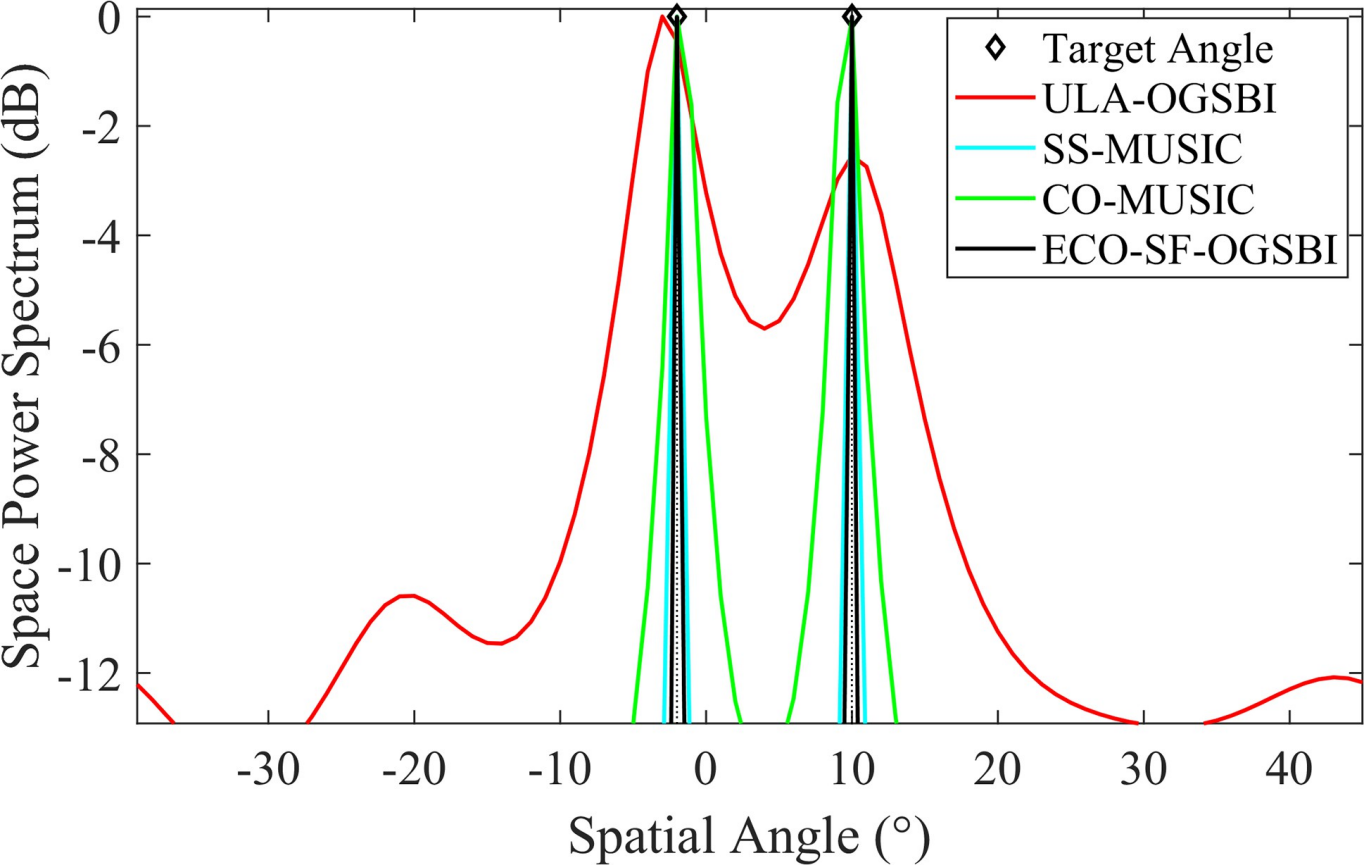
Spatial spectrum estimation for three algorithms.

**Fig 6:**
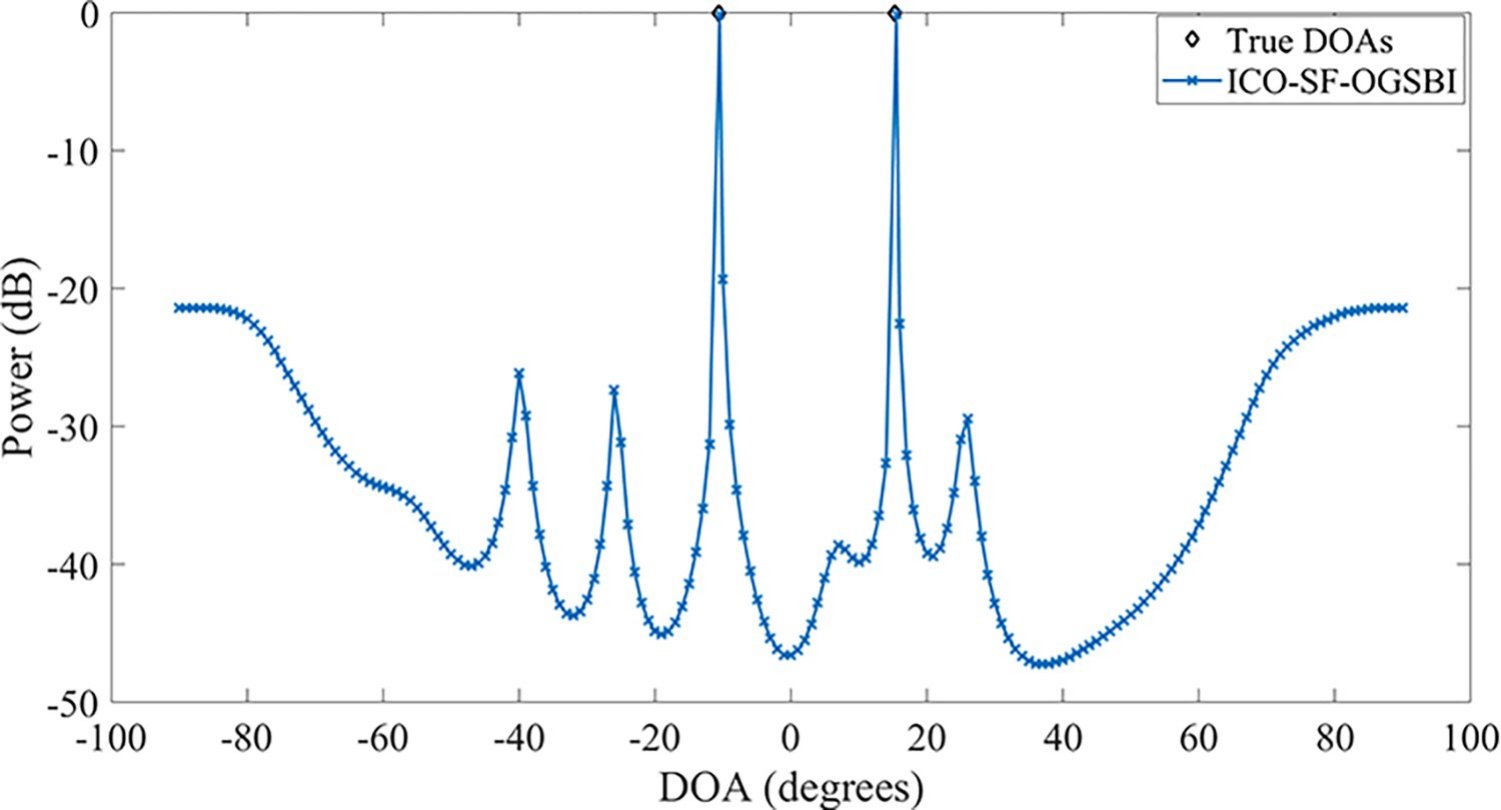
Spatial spectrum estimation at a signal-to-noise ratio of -20 dB.

Two linearly modulated frequency signal sources with incident directions in the range of [-2° 10°] are considered. The input signal-to-noise ratio (SNR) of the array element is 0 dB. Fig 5 illustrates the performance of the CO-MUSIC algorithm, the CO-SS-MUSIC algorithm, the OGSBI algorithm for a homogeneous array of 8 array elements (ULA-OGSBI), and our proposed algorithm for the same conditions on the DOA spatial spectrum estimation. The results demonstrate that the algorithm is capable of accurately detecting the angle of the two target signals, resulting in a smaller main flap width and a higher spatial-spectral gain, which closely matches the incident direction. Furthermore, the algorithm is able to reconstruct the signals using the signal subspace, which reduces the interference of noise and thus improves the accuracy of the DOA estimation at low signal-to-noise ratios. The results presented in Fig 6 demonstrate that the proposed algorithm is still capable of estimating the angle of the signal at a number of snapshots of 200 and a SNR of -20 dB.

**Fig 7:**
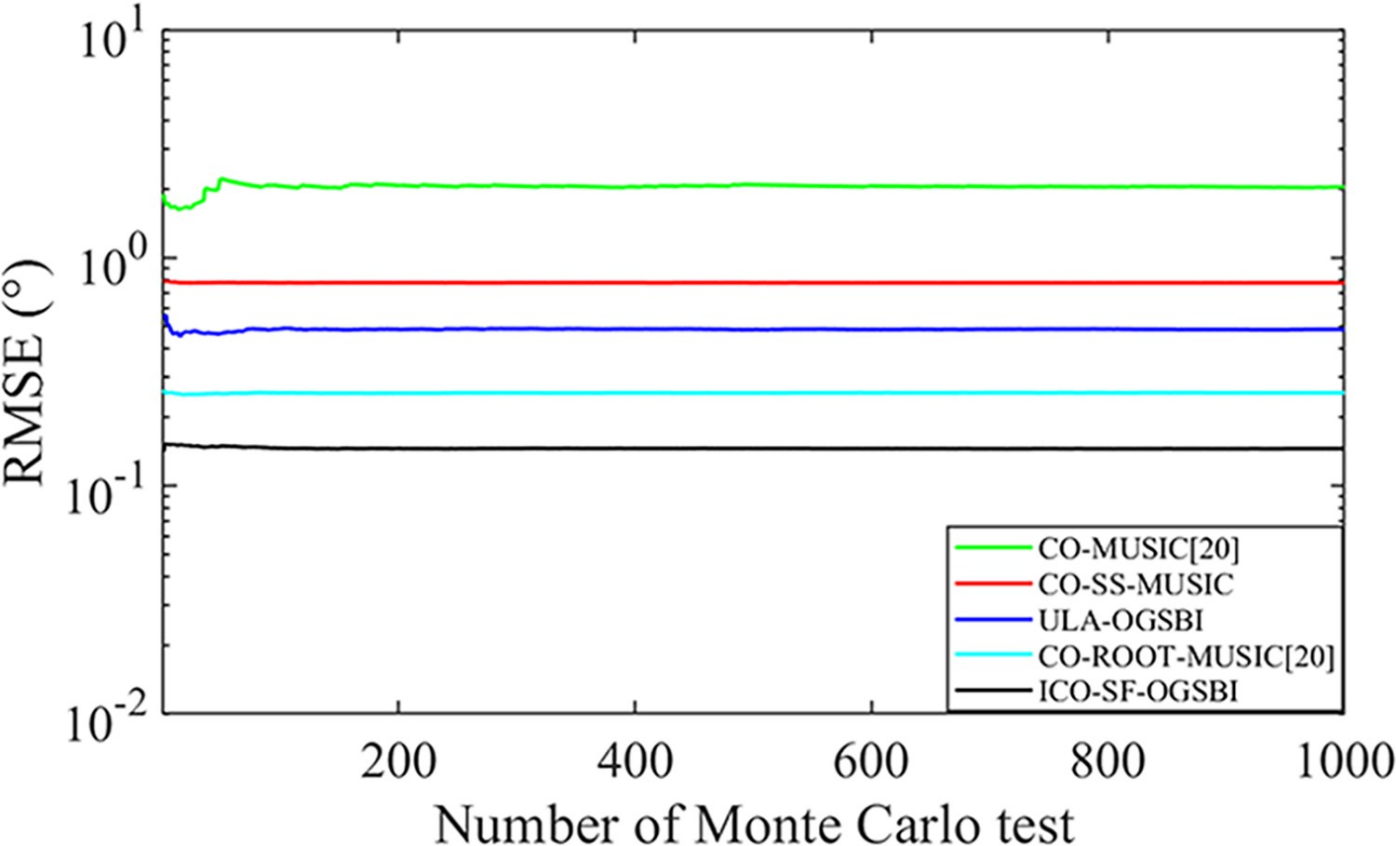
Variation of RMSE with the number of Monte Carlo experiments.

Fig 7 illustrates the variation in the RMSE value with the number of Monte Carlo experiments. In this study, Monte Carlo simulations were conducted to assess the performance of the proposed method. The root mean square error (RMSE) was observed across varying numbers of Monte Carlo trials to ensure convergence. The presentation of RMSE variation with the number of Monte Carlo experiments highlights the importance of achieving convergence. Convergence is essential for ensuring that the results are statistically reliable and not due to random sampling errors. If the number of Monte Carlo trials is insufficient, it can lead to high variance in RMSE, misrepresenting the true performance of the algorithm. The SNR is set to 0 dB, the incidence angle is in the range of [-2.56° 10.65°], and all other parameters remain unchanged. The figure clearly demonstrates this relationship. When the number of Monte Carlo iterations is less than 100, the degree of convergence of the RMSE value is low. Therefore, in order to ensure comprehensive consideration, the number of Monte Carlo experiments in this paper is selected to be 200.

**Fig 8:**
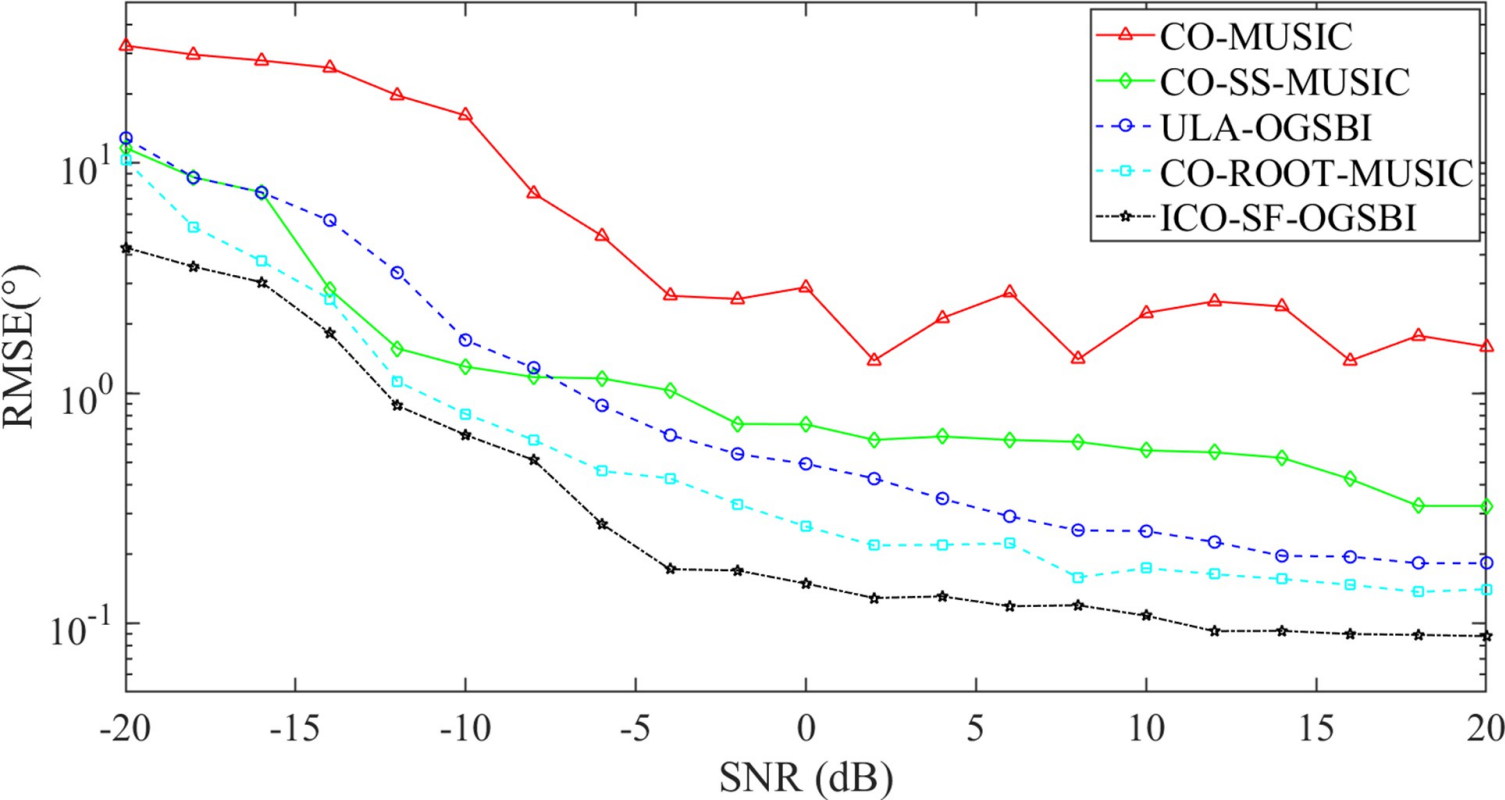
Variation of RMSE with input SNR.

Fig 8 illustrates the root mean square error of the direction-of-arrival estimation for the five algorithms as a function of the input SNR of the array element, considering two linear frequency modulation signal sources and the number of snapshots is *T* = 200. The input SNR was set at -20:2:20 dB and the incidence direction is set at [-2.56° 10.65°]. In order to ensure the reliability of the algorithm results, the results of each simulation were obtained through 200 Monte Carlo experiments. Fig 8 illustrates that the RMSE of the proposed method is low and that the estimation performance is enhanced by approximately 58.29% in comparison to the CO-ROOT-MUSIC algorithm when the SNR is -20 dB, and by 65.69% in comparison to the ULA-OGSBI algorithm. It can be concluded that the proposed algorithm exhibits superior performance in suppressing Gaussian white noise at low input SNR, effectively eliminating off-grid error at high input SNR, and demonstrating higher estimation accuracy than the other four algorithms.

**Fig 9:**
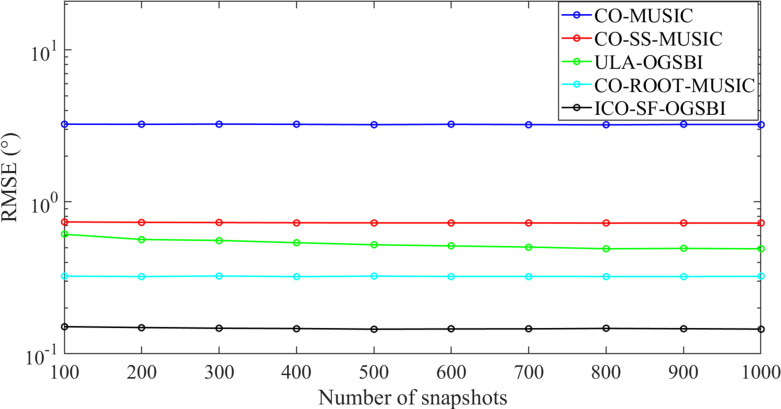
Variation of RMSE with the number of snapshots.

In order to test the effect of the snap count on the estimation accuracy of the five algorithms, two target signals were constructed. These had incidence angles of [-2.56° 10.65°], SNR = 0dB, and the snap count was adopted in the range of *T* = [100:100:1000], while the angular interval was *r* = 1°. The simulation results were obtained from 200 Monte Carlo experiments. Fig 9 illustrates the variation in the root mean square error of the DOA estimation results for the five algorithms with the number of snapshots. The analysis shows that as the number of snapshots increases, the overall RMSE of the five algorithms converges at the number of snap counts *T* = 100, indicating that the five algorithms have good DOA estimation performance at low snap counts. At a total of 200 snap counts, the estimation performance of our proposed algorithm improves by 53.85% relative to the CO-ROOT-MUSIC algorithm. The improvement is 73.66% relative to the ULA-OGSBI algorithm. In comparison to the other four algorithms, the estimation accuracy of our proposed algorithm is more precise and reliable, and its root mean square error is lower in the case of a limited number of snapshots. This effectively addresses the issue of traditional algorithms, which are significantly influenced by the number of snapshots.

### 5.3 RMSE for different grid spacing

**Fig 10:**
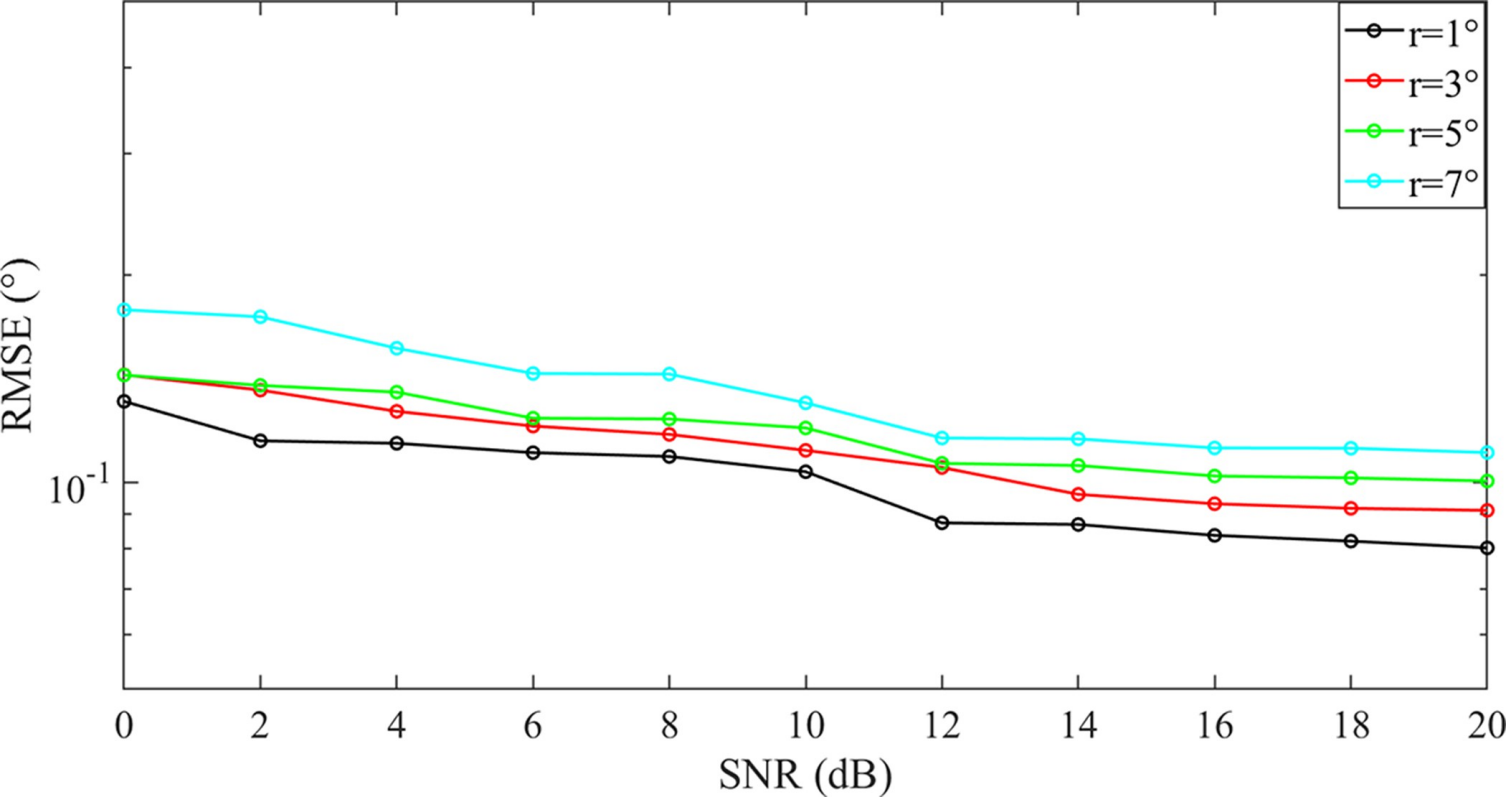
Variation of RMSE with different grid spacing.

Fig 10 shows the graph of RMSE values of the proposed algorithm as a function of SNR for grid spacing *r* = 1°, *r* = 3°, *r* = 5°, *r* = 7°. The two target orientations are [-2.56° 10.65°], the number of snapshots is 200, and the SNR = 0:2:10dB, other conditions remain unchanged. Fig 10 illustrates that the RMSE values of the four non-grid spacings exhibit a gradual decline as the SNR increases. Furthermore, it can be observed that the finer the grid spacing division, the smaller the RMSE value. It can be observed that the discrepancy in RMSE between the coarse and fine grid spacings is relatively minor, which suggests that the proposed algorithm continues to exhibit a high degree of estimation accuracy in target orientation under coarse grid spacings.

### 5.4 Close source discrimination probability

**Fig 11:**
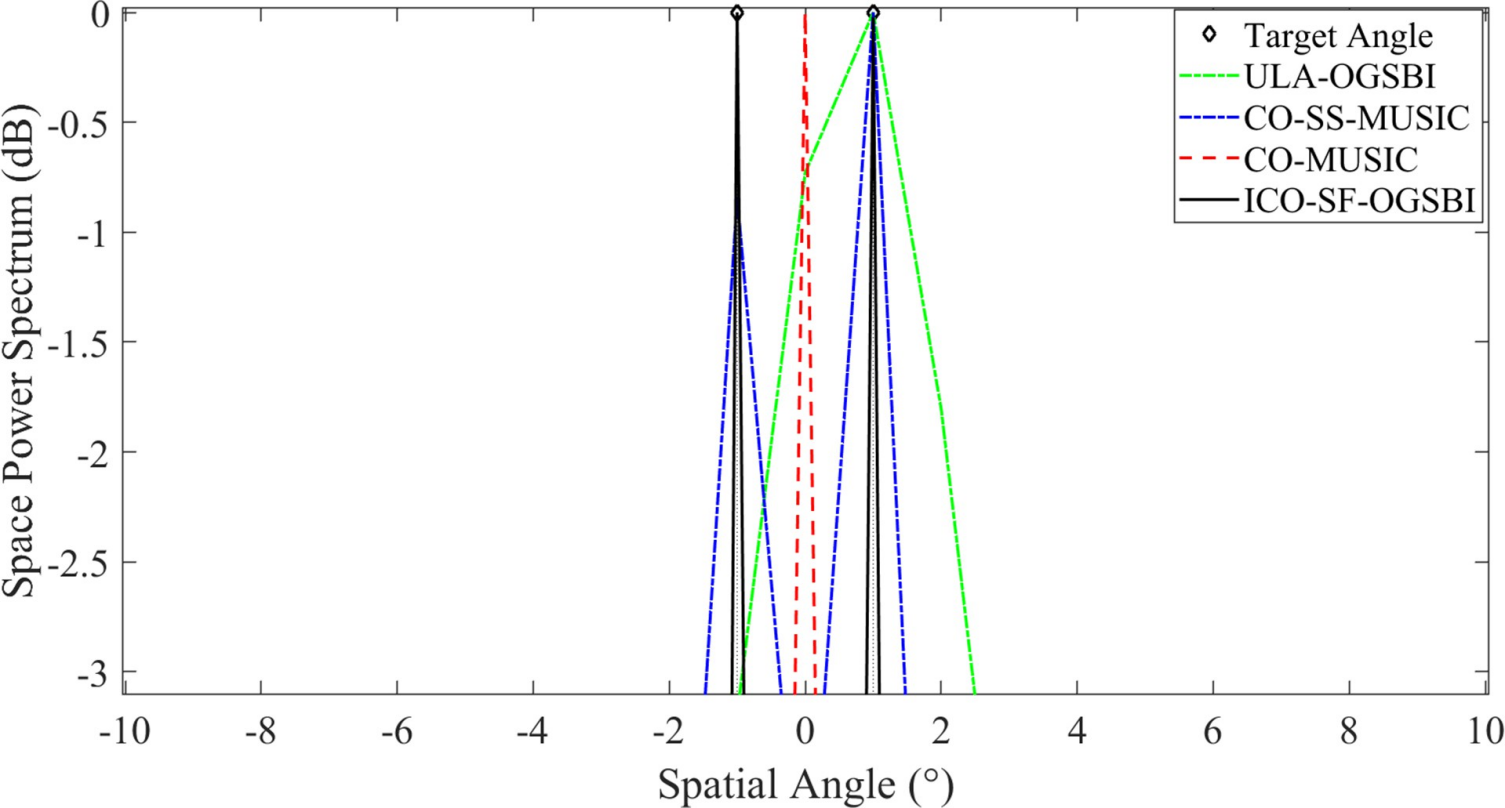
Spatial power spectrum of compact sound source.

This section is concerned with the analysis of the spatial resolution probability of tight sound sources. In order to analyze the spatial resolution ability of this paper’s algorithm for tight sound sources, let us assume that the incidence direction of the two target signals are [-1° 1°], with all other conditions remaining unchanged. The SNR is set at 5 dB, the number of snapshots is 200, and the grid spacing is *r* = 1. The spatial-spectral estimation diagrams for the three algorithms in relation to the tight sound sources are presented in Fig 11. Fig 11 illustrates that the algorithms in this paper exhibit superior spatial resolution for the tight sound sources, accompanied by narrower main flap widths and more distinct peaks. This suggests that the orientation estimation of the tight sound sources is also more accurate.

**Fig 12:**
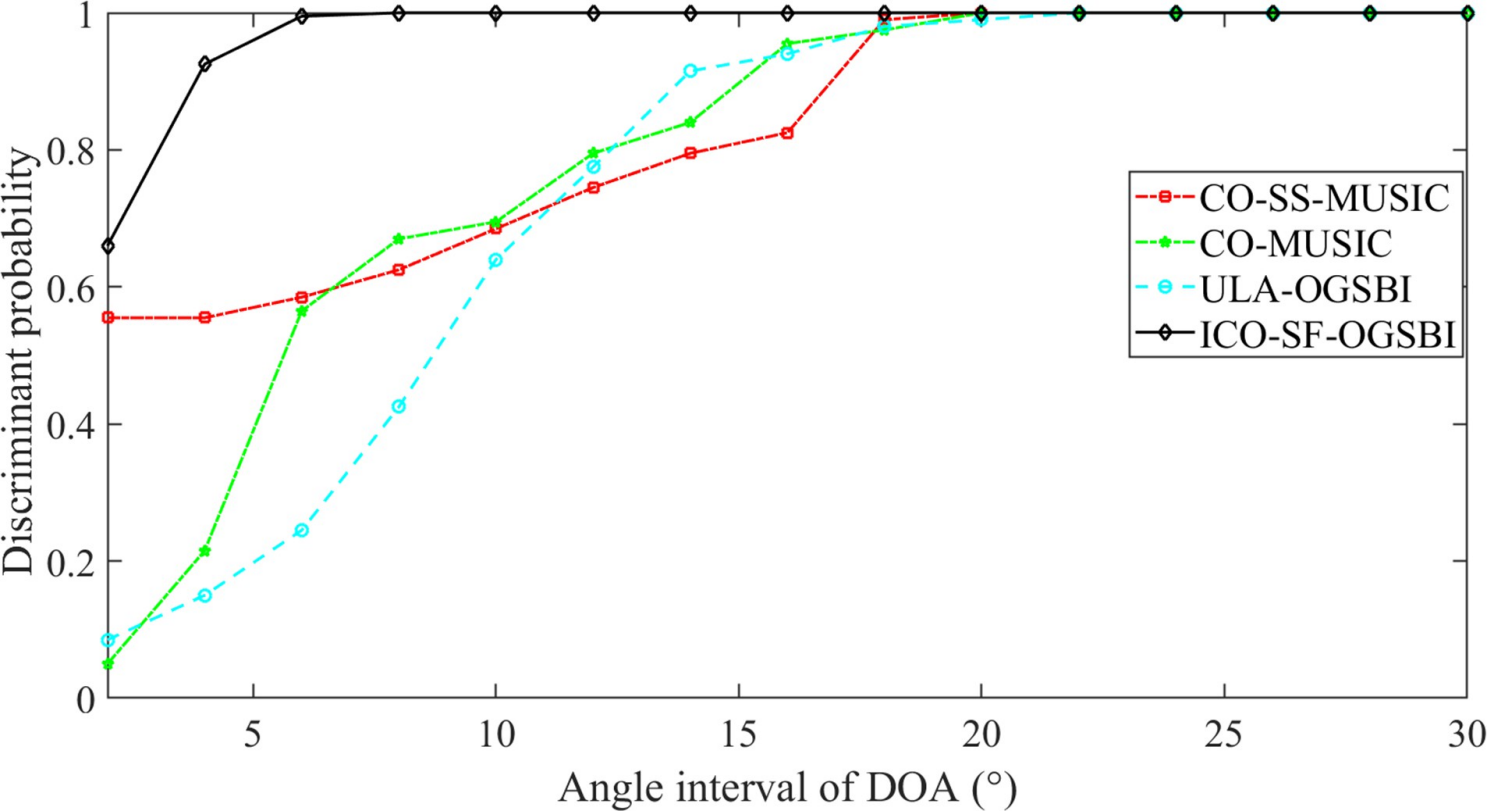
Discriminative probabilities at different DOA intervals.

In order to further investigate the discriminative ability of the proposed algorithms on the DOA estimation of spatially tightly neighboring signals, we analyze the discriminative probability of the four algorithms at different DOA intervals, with SNR = 0dB and other conditions unchanged, and the DOA of the two target signals are defined as *θ*_1_ = *m*° and *θ*_2_ = (*m*+Δ*θ*)°, and the corresponding spatial-spectral values of *ε*_1_ and *ε*_2_, respectively, and the DOA intervals are varied by 2° in steps of 2° from 2° to 30°, and 200 Monte Carlo experiments are performed at each DOA interval. 200 Monte Carlo experiments are conducted at each DOA interval, and the middle value of the two target DOAs is *θ*_*η*_ = (*θ*_1_+*θ*_2_)/2, and the corresponding spatial-spectral value of *θ*_*η*_ is *ε*_*η*_. If *ε*_*η*_≤(*ε*_1_+*ε*_2_)/2 is satisfied, then the close sound source is successfully discriminated. From Fig 12, it can be seen that the proposed algorithm achieves 100% resolution probability at the source interval of Δ*θ*≥8°. The ULA-OGSBI algorithm reaches 100% at Δ*θ*≥22°, the CO-MUSIC algorithm reaches 100% at Δ*θ*≥20° and the CO-SS-MUSIC algorithm reaches 100% at Δ*θ*≥20°. The algorithms presented in this paper demonstrate excellent spatial resolution for signals in close proximity to one another.

## 6. Sea trial data validation

**Fig 13:**
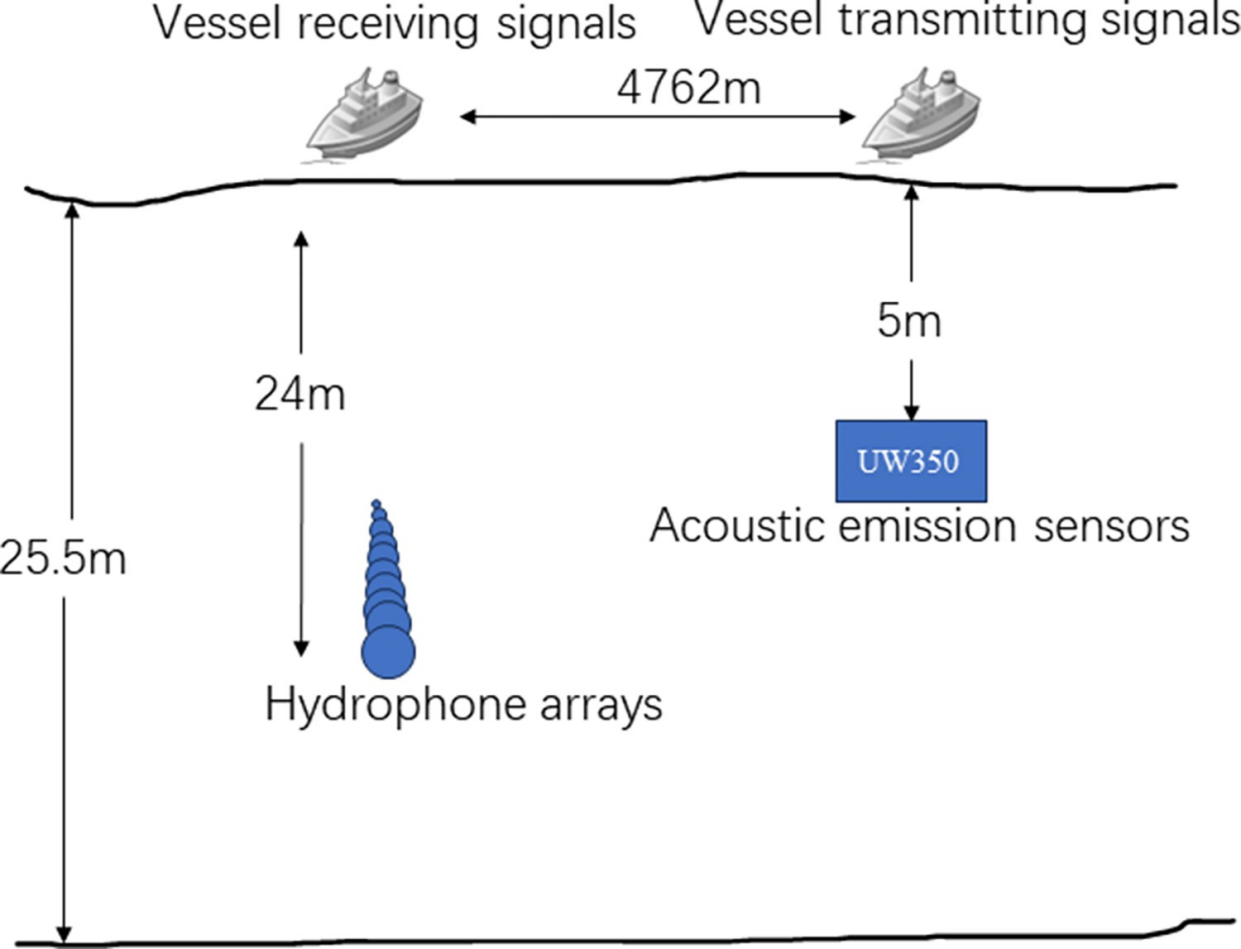
Schematic diagram of maritime array arming.

**Fig 14:**
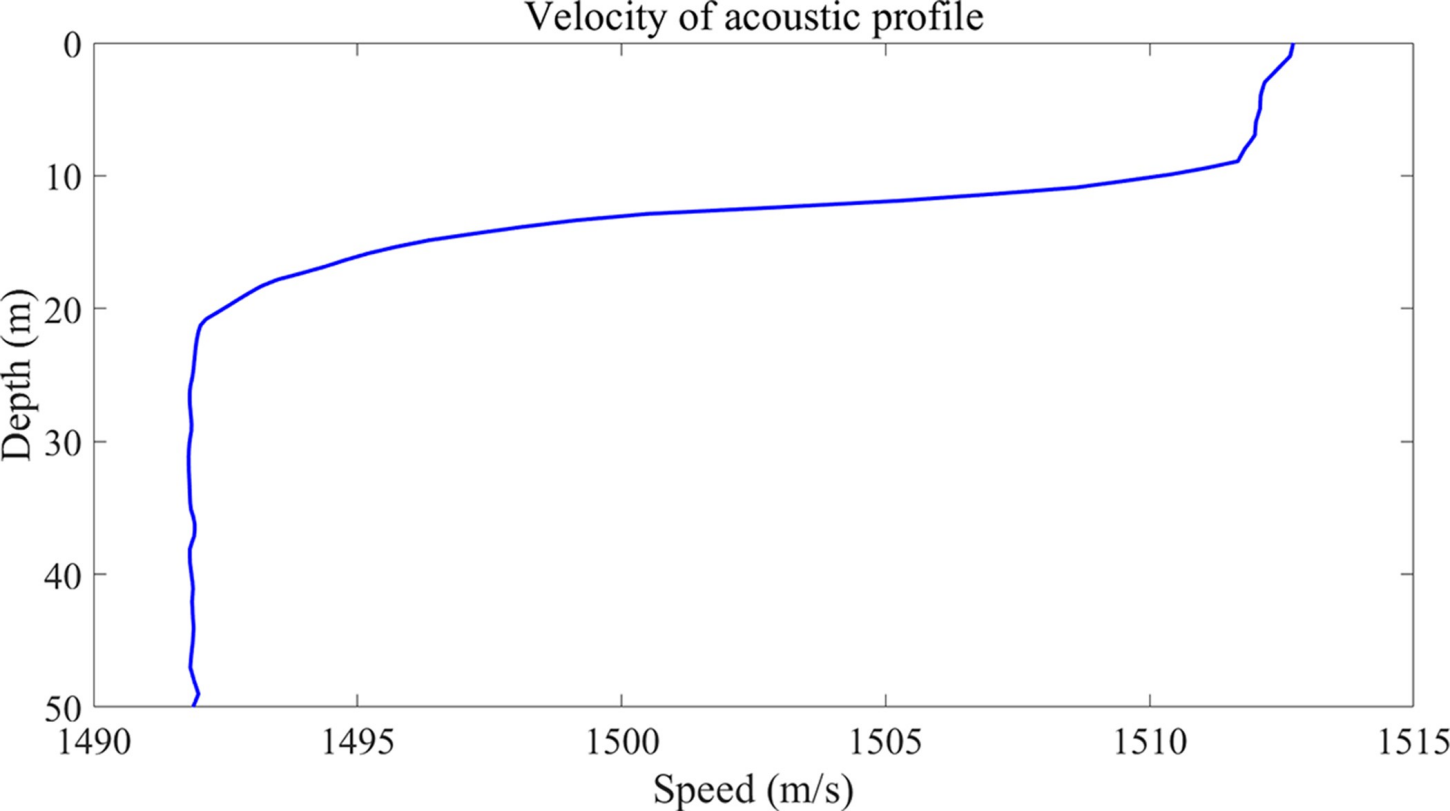
Velocity of sound profile.

The experiment was carried out in 2012 in the waters of Wangjiadao, Zhuanghe, and Dalian, with the experimental vessel acting as a local transport vessel and the two experimental vessels acting as launching and receiving vessels. On the day in question, the sea area in question was characterized by a calm sea state, with no other vessels in the vicinity and a low wind speed. In the sea test, the sound source transmitting equipment UW350 was suspended at a water depth of 5m. The transmitting signal was in the form of a 200Hz-600Hz broadband long-pulse signal, with a signal length of 3s and a sampling frequency of 10kHz. The receiving ship places the hydrophone array in a uniform manner at a depth of 24m in the seawater. The number of array elements is *M* = 13, the spacing of the array elements is half a wavelength, the hydrophone is spherical, and it is a vector hydrophone. The installation of six acoustic pressure hydrophones, each equipped with a spherical vector hydrophone, constitutes the acoustic pressure channel of the vector hydrophone. In the data validation experiments, the acoustic pressure channel of the information received by the data is the data needed for this paper. The distance between the transmitting ship and the receiving ship when the signals were transmitted, as determined by GPS data, was 4672 m. The sea depth of the experimental area, which was approximately 25.5 m, can be regarded as a flat seabed. The depth of deployment of the aforementioned equipment and the depth of seawater are measured by depth sensors with an accuracy of 0.2 percent. The schematic diagram of the deployment of the sea experiment and the measured sound speed on the same day are presented in Figs 13 and 14. Fig 14 illustrates that the speed of sound is primarily influenced by temperature and exhibits a negative gradient with depth, a characteristic of the distribution of the speed of sound in shallow seas. The sound intensity of sound waves in this distribution of the speed of sound is more attenuated.

The experimental data is generated using a 13-element uniform array. The algorithm proposed in this paper is a coprime array, which can be extended to a uniform array in the virtual domain using the interpolation method to satisfy the experimental conditions. In order to design the interpolated coprime array with *L* sensors ULA resolution, the reciprocal integers *M* and *N* are selected in such a way that max(V)≤L. It is known that the number of array elements for this experiment is 13. Therefore, a coprime array with *M* = 5 and *N* = 3 can be chosen for data validation.

**Fig 15:**
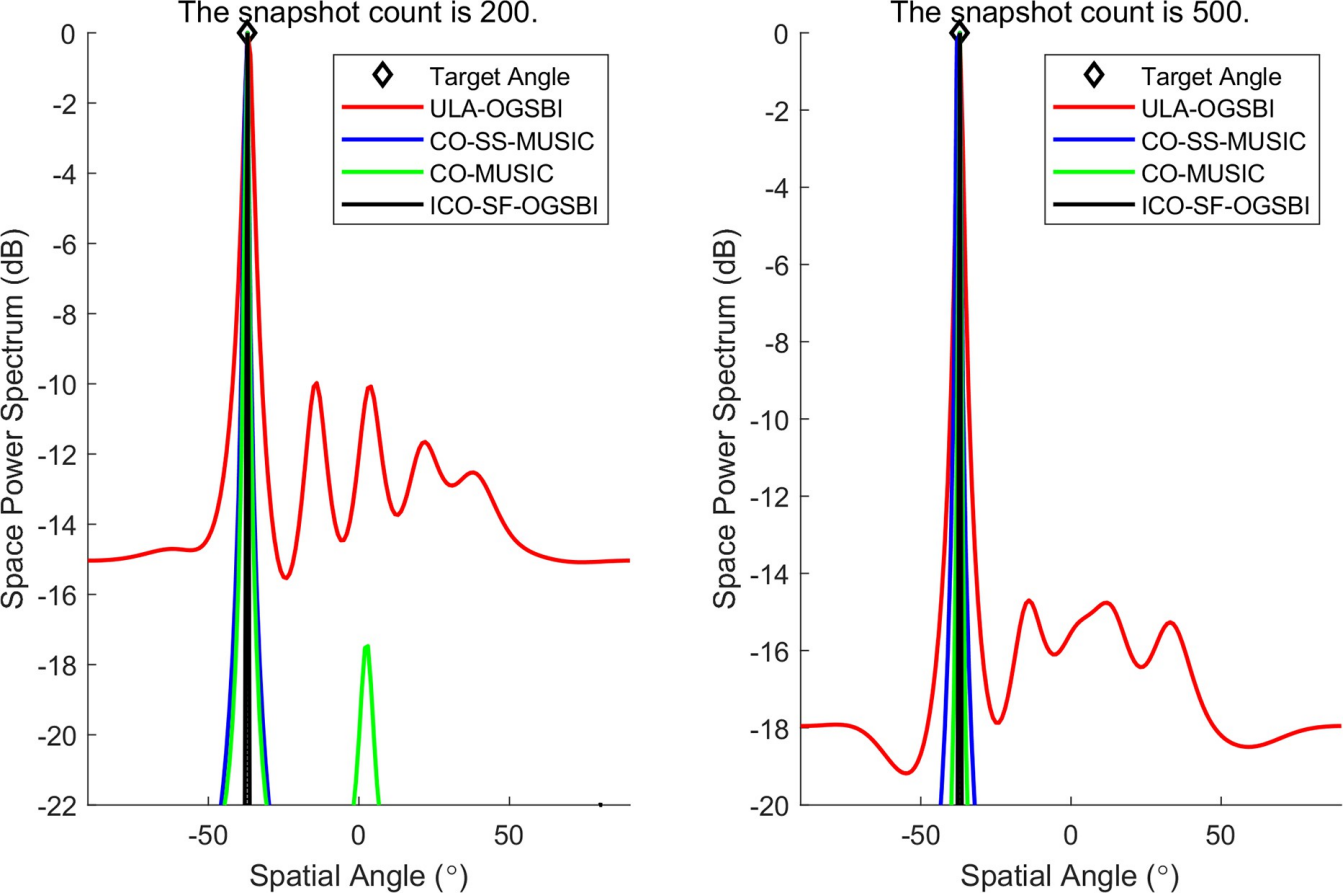
Estimation of the spatial power spectrum at the first position.

**Fig 16:**
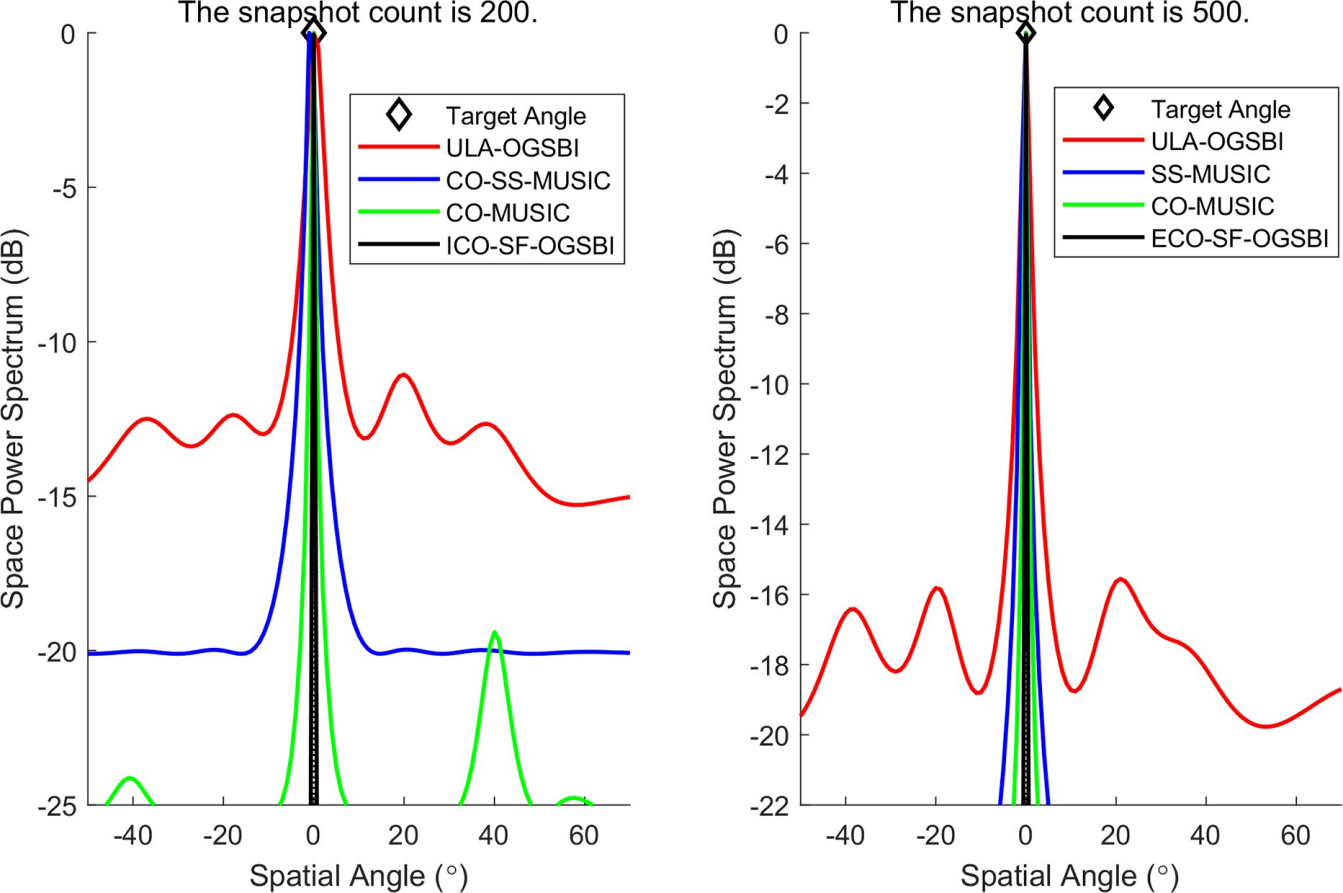
Estimation of the spatial power spectrum at the second position.

**Fig 17:**
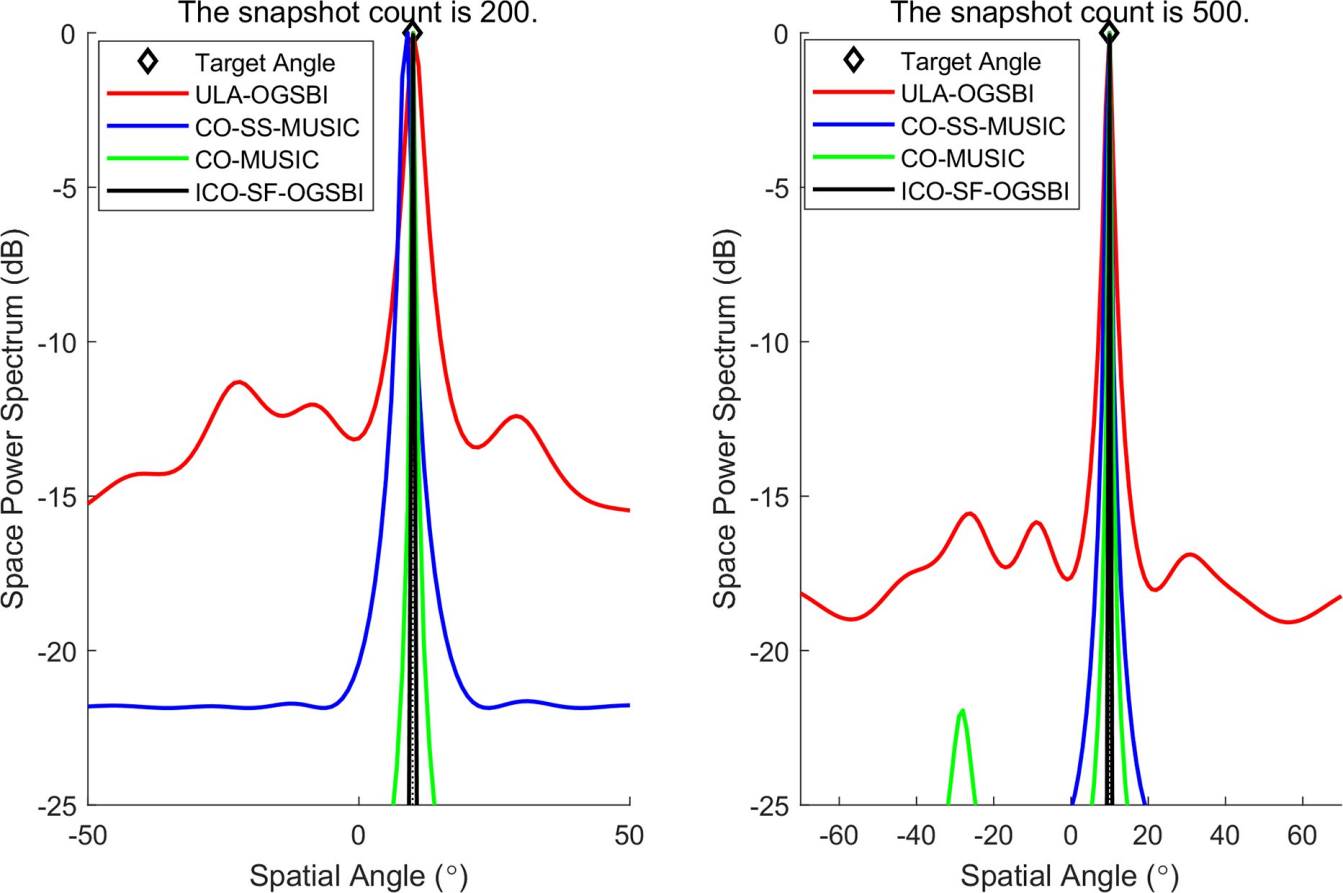
Estimation of the spatial power spectrum at the third position.

Three data sets were selected from the sea trials for the purpose of DOA validation of the target. The target orientations are given by the following values: [−37.12° 0.02° 9.89°], respectively. The results of DOA spatial spectrum estimation at different target orientations are presented in Figs 15–17. The number of snapshots selected for each orientation estimation is 100 and 500, respectively. The figures demonstrate that the CO-SS-MUSIC algorithm is capable of distinguishing the target orientation with a certain degree of accuracy. However, there is a notable deviation in the estimation of the target orientation, a wide main flap width, and a poor spatial power spectrum gain; The ULA-OGSBI algorithm is capable of approximating the target orientation. However, it has a relatively wide main flap width and there are pseudo-peaks in the spatial power spectrum, which significantly impairs the ability to discriminate of the real orientation; the CO-MUSIC algorithm can roughly estimate the target orientation with a wide main flap width and occasional serious pseudo-peaks; the algorithm presented in this paper, with a narrower main flap width and a higher spatial spectral gain, is capable of more accurately estimating the target orientation.

In order to facilitate a more accurate comparison of the performance of the four algorithms in the estimation of bearing data derived from sea trials, the experimental data from the four algorithms presented in this paper were subjected to 200 Monte Carlo experiments. The number of snapshots was selected as 100, and the mean and root mean square error of the results of the DOA estimation are presented in Table 2, which can be analyzed to show that the RMSE value of the algorithms in this paper is relatively smaller and the result of bearing estimation is more accurate. The estimation results of the algorithm in this paper are closer to the target bearing and have a stronger suppression ability to the background noise interference of the ocean, which is generally consistent with the numerical simulation analysis.

**Table 2:** Mean and root mean square errors of DOA estimate.

All kinds of algorithms	*T* = 100, *θ* = 0.02	
	Angle estimation mean	RMSE
ICO-SF-OGSBI	0.0199	0.0965
ULA-OGSBI	0.0149	0.2570
CO-SS-MUSIC	0.0148	0.2012
CO-MUSIC	-0.0226	1.1672

The results of the sea-test data validation experiments demonstrate that the method proposed in this paper can be used to estimate the DOA information received by a 13-array uniform linear array with a total of 8-array elements of the coprime array. This reduces the hardware cost. However, in practical applications, the uniform linear array constructed by using interpolation is susceptible to noise and array errors, which can result in the incorrect position of interpolated array elements. Furthermore, the fitting of the signal subspace necessitates the knowledge or accurate estimation of the energy ratio of the signal and noise. Moreover, the accurate estimation of the SNR represents a challenging problem in the actual hydroacoustic environment.

## 7. Conclusion

The aim of this study is to address the non-uniformity of the coprime array and the degradation of DOA estimation performance of traditional algorithms at low signal-to-noise ratios. In order to achieve this, the ICO-SF-OGSBI algorithm is proposed, a comparative analysis is conducted to compare and contrast the proposed algorithm with the CO-MUSIC algorithm, the CO-ROOTMUSIC algorithm, and the ULA-OGSBI algorithm. Numerical simulation analysis and experimental data validation show that the proposed algorithm can obtain higher array gain and resolution by interpolating the array elements on the virtual domain, and the background noise can be effectively suppressed by subspace fitting. When the SNR is -20 dB, the estimated performance of the proposed algorithm is enhanced by 58.29% in comparison to the CO-ROOT-MUSIC algorithm, and by 65.69% in comparison to the ULA-OGSBI algorithm. When the number of snapshots is 200, the estimation performance of the proposed algorithm is 53.85% better than that of the CO-ROOT-MUSIC algorithm, and 73.66% better than that of the ULA-OGSBI algorithm. In comparison to the conventional algorithm, the algorithm presented in this paper is less susceptible to variations in grid spacing and the number of snapshots, exhibiting robust performance. It is capable of accurately estimating both angles of [-1° 1°] when resolving tight sources and can achieve 100% resolution at Δ*θ*≥8°. The proposed algorithm not only effectively addresses the issues of low accuracy in target direction-of-arrival estimation under a low signal-to-noise ratio and the application of a non-uniform array to traditional algorithms, but also provides a theoretical foundation for the research of underwater target direction-of-arrival estimation.
